# *Piezo1* haploinsufficiency does not alter mechanotransduction in mouse cochlear outer hair cells

**DOI:** 10.14814/phy2.12701

**Published:** 2016-02-11

**Authors:** Laura F. Corns, Walter Marcotti

**Affiliations:** ^1^ Department of Biomedical ScienceUniversity of SheffieldSheffieldUK

**Keywords:** Cochlea, electrophysiology, hair cell, mechanoelectrical transduction, Piezo1

## Abstract

The mechanoelectrical transducer (MET) channels located at the stereocilia tip of cochlear hair cells are crucial to convert the mechanical energy of sound into receptor potentials, but the identity of its pore‐forming subunits remains uncertain. *Piezo1*, which has been identified in the transcriptome of mammalian cochlear hair cells, encodes a transmembrane protein that forms mechanosensitive channels in other tissues. We investigated the properties of the MET channel in outer hair cells (OHCs) of *Piezo1* mice (postnatal day 6–9). The MET current was elicited by deflecting the hair bundle of OHCs using sinewave and step stimuli from a piezo‐driven fluid jet. Apical and basal OHCs were investigated because the properties of the MET channel vary along the cochlea. We found that the maximal MET current amplitude and the resting open probability of the MET channel in OHCs were similar between *Piezo1* ^+/−^ haploinsufficient mice and wild‐type littermates. The sensitivity to block by the permeant MET channel blocker dihydrostreptomycin was also similar between the two genotypes. Finally, the anomalous mechano‐gated current, which is activated by sheer force and which is tip‐link independent, was unaffected in OHCs from *Piezo1* ^+/−^ haploinsufficient mice. Our results suggest that Piezo1 is unlikely to be a component of the MET channel complex in mammalian cochlear OHCs.

## Introduction

The hair cells of the organ of Corti within the mammalian cochlea convert mechanical stimuli into receptor potentials. The hair bundle atop each cell contains multiple rows of stereocilia of increasing height, which are deflected in response to sound. The stereocilia are connected by tip links in the direction of optimal mechanosensitivity (Pickles et al. 1984; Schwander et al. 2010), such that their deflections toward the tallest stereocilia elicit excitatory responses. The mechanoelectrical transducer (MET) channel complex, which allows the influx of positive ions into the hair cells, resides in the region where the tip link inserts into the tip of the lower stereocilia (Beurg et al. 2009). In recent years, four molecules have been identified as likely components of the MET channel complex: TMHS/LHFPL5 (Xiong et al. 2012), TMIE (Zhao et al. 2014), TMC1 (Kawashima et al. 2011; Pan et al. 2013; Kurima et al. 2015), and TMC2 (Kawashima et al. 2011; Kurima et al. 2015). While TMHS/LHFPL5 and TMIE are unlikely to form the pore‐forming subunits of the MET channel (Xiong et al. 2012; Zhao et al. 2014), mutations in *tmc1* and *tmc2* have been shown to affect the MET channel (Kawashima et al. 2011; Pan et al. 2013; Corns et al. 2016). The possibility that other proteins could be contributing to the MET channel pore in hair cells has been suggested by the fact that an anomalous MET current, which is independent of tiplinks and is instead evoked by sheer stress (Marcotti et al. 2014), has been shown to be present in mice lacking both TMC1 and TMC2 (Beurg et al. 2014).

In this study, we investigated Piezo1, which has been shown to constitute the pore‐forming subunit of some mechanosensitive channels (Coste et al. 2010, 2012). A transcriptome analysis has demonstrated that *Piezo1* (*Fam38A)* is expressed in mammalian cochlear hair cells (Liu et al. 2014), suggesting that it could play a role in mechanosensory transduction in these cells. Although homozygous mutant (*Piezo1* ^−/−^) mice are embryonic lethal (Li et al. 2014; Ranade et al. 2014a), *Piezo1* ^+/−^ mice show haploinsufficiency, which affects the coupling between sheer stress and Ca^2+^ entry into endothelial cells (Li et al. 2014). In cochlear OHCs, we found no differences in maximal MET current amplitude, reversal potential, adaptation, or blocking potency of DHS between *Piezo1* ^+/−^ and *Piezo* ^+/+^ mice. Moreover, the anomalous MET current (Marcotti et al. 2014) was still present in *Piezo1* ^+/−^ OHCs.

## Methods

### Animals and genotyping

All experiments were performed in accordance with Home Office regulations under the Animals (Scientific Procedures) Act 1986 and following approval by the University of Sheffield Ethical Review Committee. *Piezo1* ^+/−^ mice were obtained from David J. Beech (University of Leeds, Leeds, UK) and maintained on a C57BL/6J background. Genotype was determined by PCR analysis of DNA from tail clips (Li et al. 2014). Recordings were made from mice of either sex.

### Hearing test

To test whether the hearing in *Piezo1* haploinsufficient mice was affected, we performed a visual observation of the Preyer reflex, which is a back‐flick of the pinna in response to sound. Control and haploinsufficient mice were placed in a large plastic box and subjected to a 20 kHz tone burst with an intensity of 95 dB sound pressure level from distances of 20 cm above the head, using a custom‐built device provided by the Medical Research Council Institute of Hearing Research (Nottingham, UK).

### Tissue preparation

To study OHCs in acutely dissected organs of Corti, mice were killed by cervical dislocation, the cochlea removed and the organ of Corti dissected in extracellular solution composed of (in mmol/L): 135 NaCl, 5.8 KCl, 1.3 CaCl_2_, 0.9 MgCl_2_, 0.7 NaH_2_PO_4_, 5.6 d‐glucose, 10 HEPES‐NaOH, 2 Na‐pyruvate. Amino acids and vitamins (Eagle's MEM) were added from concentrates (pH 7.5, 308 mOsm/kg). Once dissected, the apical and basal coils of the organ of Corti were transferred to a microscope chamber containing extracellular solution and viewed on a Leica DMLFS microscope (Leica Micro Systems, Wetzlar, Germany) through a long working‐distance 63× water‐immersion objective.

### Whole‐cell patch clamp

Recordings were made from postnatal day 6 (P6) to P9 OHCs at room temperature (20–25°C) using an Optopatch amplifier (Cairn Research Ltd, Faversham, UK). Patch pipettes with a typical resistance of 2–4 MΩ were pulled from soda glass capillaries. In order to reduce the fast electrode capacitative transient, the shank of each capillary was coated with surf wax (Mr Zoggs Sex Wax, CA). Pipettes were filled with an intracellular solution of composition (in mmol/L): 106 l‐glutamic acid, 20 CsCl, 10 Na_2_‐phosphocreatine, 3 MgCl_2_, 1 EGTA‐CsOH, 5 Na_2_ATP, 5 HEPES, and 0.3 GTP (adjusted to pH 7.28 with 1 mol/L CsOH; 294 mOsm/kg). An l‐glutamic acid‐based intracellular solution was used as it preserves cellular ultrastructure and improves the stability of recordings (Kay 1992). A similar solution has extensively been used for investigating the biophysical properties of mammalian cochlea hair cells (e.g., Moser and Beutner 2000; Corns et al. 2014). Data acquisition was performed using pClamp software (Molecular Devices, Sunnyvale, CA) using a Digidata 1440A. Data were filtered at 5 kHz (8‐pole Bessel). Offline data analysis was performed using Origin software (OriginLab, Northampton, MA). Membrane potentials were corrected for a liquid junction potential of −11 mV measured between electrode and bath solution.

### Hair bundle stimulation

Mechanoelectrical transducer currents were elicited using a fluid jet from a pipette driven by a 25‐mm diameter piezoelectric disc (Kros et al. 1992; Corns et al. 2014). The fluid jet pipette tip had a diameter of 8–10 *μ*m and was positioned at about 8 *μ*m from the hair bundles, which elicited a maximal MET current. Mechanical stimuli were applied as steps or 50 Hz sinusoids (filtered at 1 kHz, 8‐pole Bessel). The tiplink independent anomalous current was elicited by applying unphysiologically strong mechanical stimuli causing a sheer force displacement of the cuticular plate of OHCs (Marcotti et al. 2014). The conversion value used to express the piezo‐driver voltage into bundle displacement during normal fluid jet stimulation was as previously reported when using the above standard recording conditions (10 nm/V: Corns et al. 2014).

### Dihydrostreptomycin application

In order to test the effects of the MET channel blocker dihydrostreptomycin (DHS, Sigma, Gillingham, UK) on the MET current, OHCs were superfused with a normal extracellular solution containing 10 *μ*mol/L DHS, which in these cells represents its half‐blocking concentration near −80 mV (Marcotti et al. 2005). The stock solution of DHS (100 mmol/L: molecular weight = 730.7) was prepared using extracellular solution (see above). During the recordings, the DHS test solution was present in the fluid jet and also superfused via a pipette positioned orthogonally to the axis of mechanical sensitivity of the hair bundle. To ensure complete exchange of the normal extracellular solution with that containing DHS, the test solution was sucked into the fluid jet prior to stimulation of the hair bundles.

### Statistical analysis

Statistical comparisons of means were made by one‐way ANOVA, with *P *<* *0.05 selected as the criterion for statistical significance. All values are quoted as mean ± SEM.

## Results

Hearing in *Piezo1* haploinsufficient mice was visually tested by probing for the presence of a Preyer's reflex in response to a 20 kHz tone burst. Although the *Piezo1* ^+/−^ mice responded similarly to littermate controls (*n *=* *3 each genotype; >3 months of age), a Preyer's reflex test cannot detect less severe forms of sensorineural deafness (Jero et al. 2001). In addition, the absence of a hearing phenotype would not preclude the involvement of Piezo1 in mechano‐electrical transduction since, for example, the loss of TMC2 does not confer hearing loss despite affecting the MET current in the immature cochlear hair cells (Kawashima et al. 2011).

**Figure 1:**
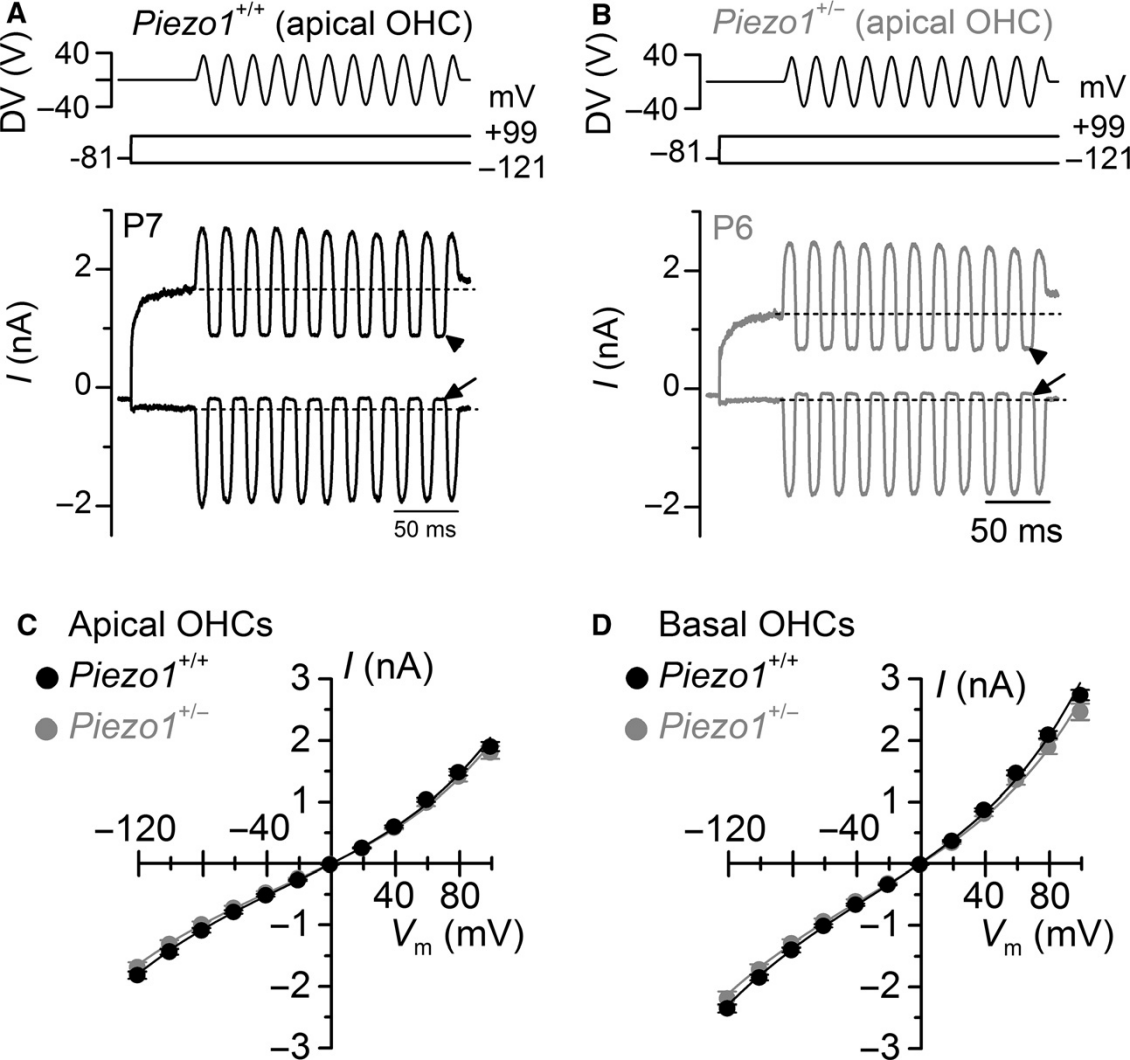
Maximal amplitude of the mechanoelectrical transducer (MET) current in outer hair cells (OHCs) is not affected in *Piezo1* ^*+/−*^ haploinsufficient mice. Saturating MET currents in apical OHCs from *Piezo1* ^+/+^ (A, P7) and *Piezo1* ^+/−^ (B, P6) mice in response to 50 Hz sinusoidal force stimuli to the hair bundles at membrane potentials of −121 and +99 mV. Driver voltage (DV) stimuli to the fluid jet are shown above the traces, with positive deflections of the DV being excitatory. The arrows and arrowheads indicate the closure of the transducer channel in response to inhibitory bundle stimuli at −121 and +99 mV, respectively. Average peak to peak MET current–voltage curves from apical (C) and basal (D) OHCs of *Piezo1* ^+/+^ (10 apical and 8 basal OHCs) and *Piezo1* ^+/−^ (16 apical and 7 basal OHCs) mice. Recordings were obtained by mechanically stimulating the hair bundles of OHCs while stepping their membrane potential from −121 to +99 mV in 20 mV increments.

To investigate the possible involvement of Piezo1 in hair cells, MET currents in apical and basal OHCs were elicited by displacing stereociliary hair bundles with sinewave stimuli from a piezoelectric fluid jet. A positive driver voltage to the fluid jet pushed solution out, deflecting the hair bundles in the excitatory direction toward the tallest stereocilia. At negative membrane potentials, large inward MET currents were recorded in all OHCs tested (Fig. 1A, B). At a membrane potential of −121 mV, the maximum amplitude of the MET current in apical OHCs was similar between *Piezo1* ^+/+^ (−1812 pA ± 61, *n *=* *10) and *Piezo1* ^+/−^ (−1692 pA ± 84; *n *=* *16) mice. The MET current amplitude normally increases from the low‐frequency OHCs in the apex to the high‐frequency cells in the base (Ricci 2002; Johnson et al. 2011; Kim and Fettiplace 2013). We found that the maximal MET current in basal OHCs was similar between the two genotypes (*Piezo1* ^+/+^: −2351 pA ± 69, *n *=* *8; *Piezo1* ^+/−^: −2189 pA ± 113; *n *=* *7), and it was significantly larger (*P *<* *0.001) than that recorded in apical OHCs in both *Piezo1* ^+/+^ and *Piezo1* ^+/−^ mice. As the membrane potential was stepped to more depolarized values, the inward MET current decreased in size at first and then reversed to become an outward current (Fig. 1). The reversal potential of the MET current did not vary between apical OHCs from *Piezo1* ^+/+^ (−1.3 mV ± 0.7, *n *=* *10) and *Piezo1* ^+/−^ (−1.8 mV ± 0.4, *n *=* *16) mice (Fig. 1C), or between basal OHCs (*Piezo1* ^+/+^: −3.2 mV ± 0.4, *n *=* *8; *Piezo1* ^+/−^: −3.4 mV ± 0.5, *n *=* *7, Fig. 1D).

The resting MET current, which is defined as the current flowing into the channel in the absence of bundle stimulation, can be measured from the difference between the holding current and the current observed during inhibitory bundle stimulation, that is, when negative driver voltages pull solution into the fluid jet and completely close the MET channel. The resting MET current was represented as a proportion of the total MET current (i.e., resting open probability, *P*_o_). At the negative membrane potentials of −121 mV, the resting *P*_o_ of apical (0.077 ± 0.008, *n *=* *16) and basal (0.078 ± 0.012, *n *=* *7) OHCs from *Piezo1* ^+/−^ mice was similar to that observed in OHCs from their wild‐type littermates (*Piezo1* ^+/+^ apical: 0.076 ± 0.006, *n *=* *10; basal: 0.067 ± 0.004, *n *=* *8), and in agreement with previous finding in normal wild‐type mice (Géléoc et al. 1997; Stauffer and Holt 2007; Corns et al. 2014). The resting *P*_o_ of the MET channel in wild‐type mice normally increases when stepping the membrane potential to positive values near the Ca^2+^ equilibrium potential (*Piezo1* ^+/+^ apical: 0.40 ± 0.04, *n *=* *10; basal: 0.30 ± 0.03, *n *=* *8; see also Fig. 1A). This is because while increasing Ca^2+^ influx through the MET channels promotes adaptation, thus closing some channels, reducing its influx increases the channel *P*_o_ (Assad et al. 1989; Crawford et al. 1991; Ricci et al. 1998; Corns et al. 2014). Similar increases in *P*_o_ were also observed in OHCs from *Piezo1* ^+/−^ mice (apical: 0.38 ± 0.04, *n *=* *16; basal: 0.33 ± 0.03, *n *=* *7; see also Fig. 1B).

**Figure 2:**
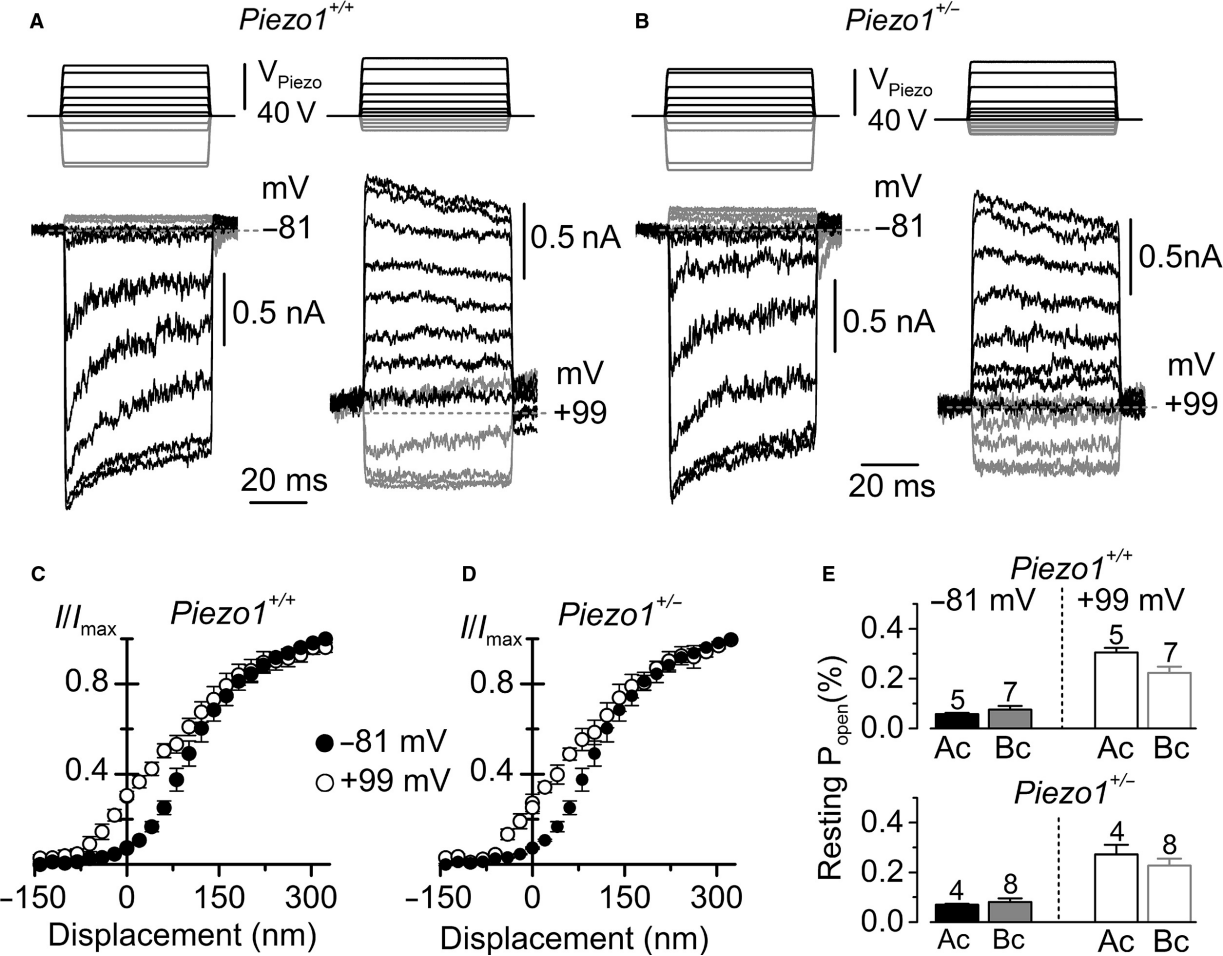
Mechanoelectrical transducer (MET) current adaptation in apical or basal outer hair cells (OHCs) is similar between *Piezo1* ^+/+^ and *Piezo1* ^+/−^ mice. A and B, Force step stimuli of 50 ms duration (top) elicited MET currents (bottom panels). Recordings were obtained at −81 mV (left panels) and +99 mV (right panels) from apical OHCs of *Piezo1* ^+/+^ (A) and *Piezo1* ^+/−^ (B) mice. At −81 mV, positive driver voltages (excitatory stimuli) elicited large inward currents that adapted or decline over time for intermediate stimuli for both *Piezo1* ^+/+^ and *Piezo1* ^+/−^ OHCs; in the inhibitory direction (negative driver voltages) the step stimuli closed the small MET current that was available at rest (gray traces). At +99 mV (right panels), adaptation was absent and the resting MET current was increased in both genotypes. C and D, Normalized peak MET current recorded from apical OHCs of P7 *Piezo1* ^+/+^ (*n *=* *5) and P7–P8 *Piezo1* ^+/−^ (*n *=* *4) mice, respectively. The MET currents were recorded at the holding potential of −81 and +99 mV from the same OHCs and plotted as a function of bundle displacement. The Ca^2+^‐dependent adaptive leftward shift of the MET current at +99 mV was normal in OHCs from both genotypes. E, Resting *P*_open_ at −81 and +99 mV obtained from apical (Ac) and basal (Bc) coil OHCs in the two different genotypes.

To investigate further the adaptation properties of the MET current in *Piezo1* ^+/−^ mice, the hair bundles of OHCs were stimulated with step stimuli. While holding the cell at −81 mV, small nonsaturating bundle displacements in the excitatory direction caused a time‐dependent decline of the MET current (e.g., MET current adaptation) in both *Piezo1* ^+/+^ and *Piezo1* ^+/−^ OHCs (Fig. 2A, B: left panels). Adaptation is a crucial property of the MET channel allowing the resetting of its operating range (Crawford et al. 1991; Fettiplace and Hackney 2006). Adaptation was similar between the two genotypes, exhibiting a fast time constant (*Piezo1* ^+/+^: 1.35 ± 0.57 ms, *n *=* *4; *Piezo1* ^+/−^: 1.02 ± 0.12 ms, *n *=* *4) and a slow time constant (*Piezo1* ^+/+^: 21 ± 7 ms; *Piezo1* ^+/−^: 20 ± 5 ms). MET current adaptation was lost and the channel resting *P*_o_ increased on stepping the membrane potential to +99 mV (Fig. 2A, B: right panels), a condition that prevents or strongly reduces Ca^2+^ entry via the MET channels. As for the sinewave stimuli, the *P*_o_ of the MET current at −81 and +99 mV did not differ between wild type and *Piezo1* ^+/−^ in both apical and basal coil OHCs (Fig. 2E).

**Figure 3:**
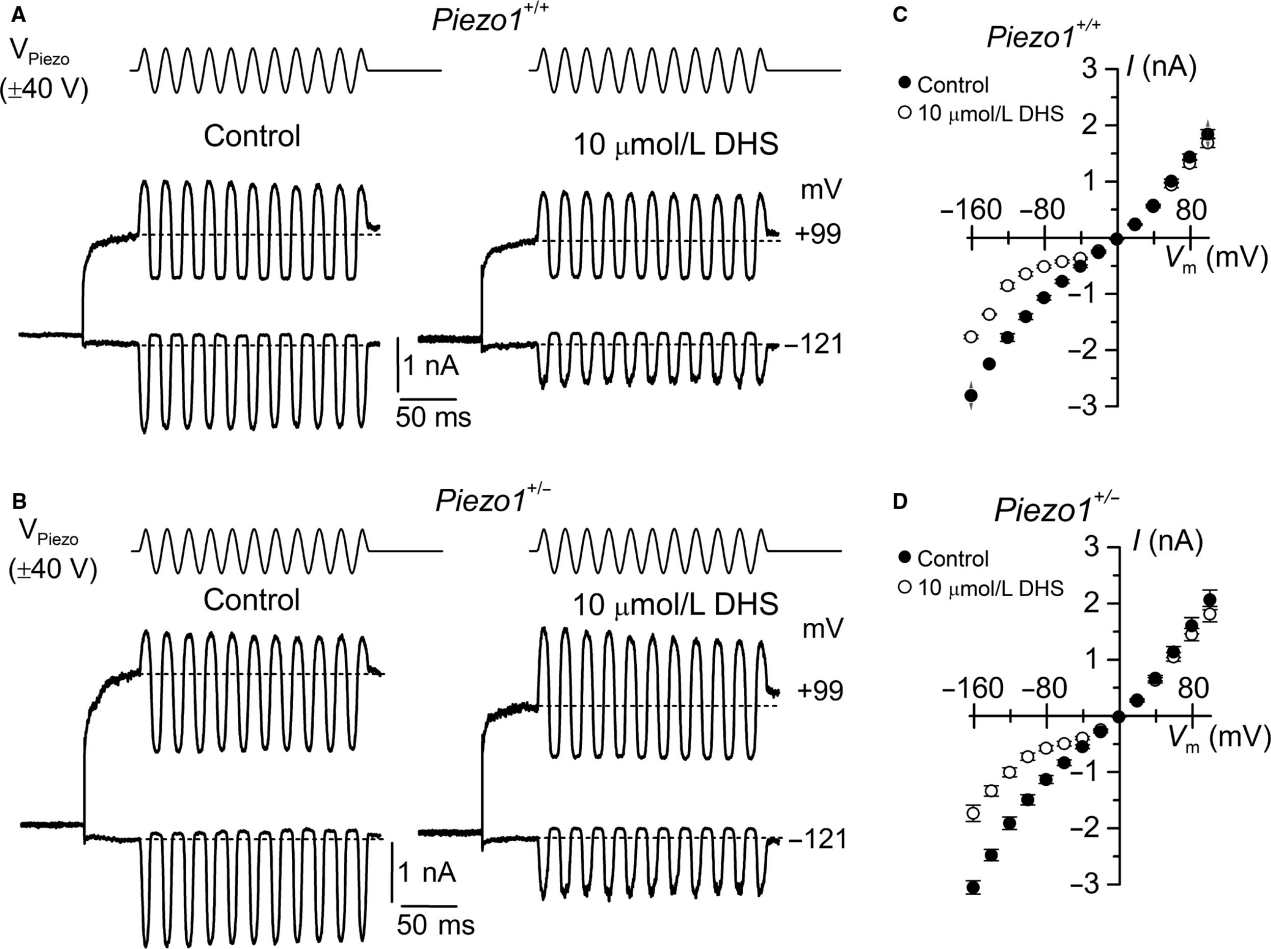
The mechanoelectrical transducer (MET) current in *Piezo1* ^+/−^ and *Piezo1* ^+/+^ outer hair cells (OHCs) is similarly reduced by the channel blocker dihydrostreptomycin. A and B, Saturating MET currents recorded from apical OHCs of *Piezo1* ^+/+^ (A, P7) and *Piezo1* ^+/−^ (B, P8) mice elicited by fluid jet stimulation to the hair bundles (top) superimposed to voltage steps between −161 and +99 mV in 20 mV increments. For clarity, only voltage steps of −121 and +99 mV are shown. Recordings were performed before (left panels) and during (right panels) the extracellular application of 10 *μ*mol/L dihydrostreptomycin (DHS). C and D, Average saturating MET current–voltage curves for OHCs from the apical coil of P7 *Piezo1* ^+/+^ (C, *n *=* *8) and P7–P8 *Piezo1* ^+/−^ (D, *n *=* *6) mice. Note the relief of the block at the most negative potentials.

We then investigated whether the MET current in *Piezo1* ^+/−^ mice was affected by the aminoglycoside dihydrostreptomycin (DHS), a large polycation known to block (Ohmori 1985; Kroese et al. 1989; Ricci 2002; Marcotti et al. 2005) and permeate (Marcotti et al. 2005) the MET channel in vertebrate hair cells. Using 50 Hz sinusoidal stimulation, we recorded the MET current by stepping the membrane between −161 and +99 mV in 20 mV increments from OHCs of both *Piezo1* ^+/+^ and *Piezo1* ^+/−^ mice before (Fig. 3A, B, left panels) and during the superfusion of 10 *μ*mol/L extracellular DHS (Fig. 3A, B, right panels). The peak‐to‐peak MET current–voltage curves revealed that, as previously described (Marcotti et al. 2005), extracellular DHS caused a voltage‐dependent block of the MET current with positive membrane potentials relieving the block in both genotypes (Fig. 3C, D). The block of the MET current by DHS at negative membrane potentials was partially relieved for values negative to about −80 mV, indicating that the drug was pushed from its binding site and forced through the channel pore into the cytoplasm when sufficient electrical driving force was applied (Marcotti et al. 2005). When the MET current amplitude in the presence of DHS was normalized to that recorded during control conditions (*I*_DHS_/*I*_control_) in the same OHCs and at −81 mV, we found a similar level of DHS block between *Piezo1* ^+/+^ (0.48 ± 0.01, *n *=* *8) and *Piezo1* ^+/−^ (0.50 ± 0.02, *n *=* *6) mice.

**Figure 4:**
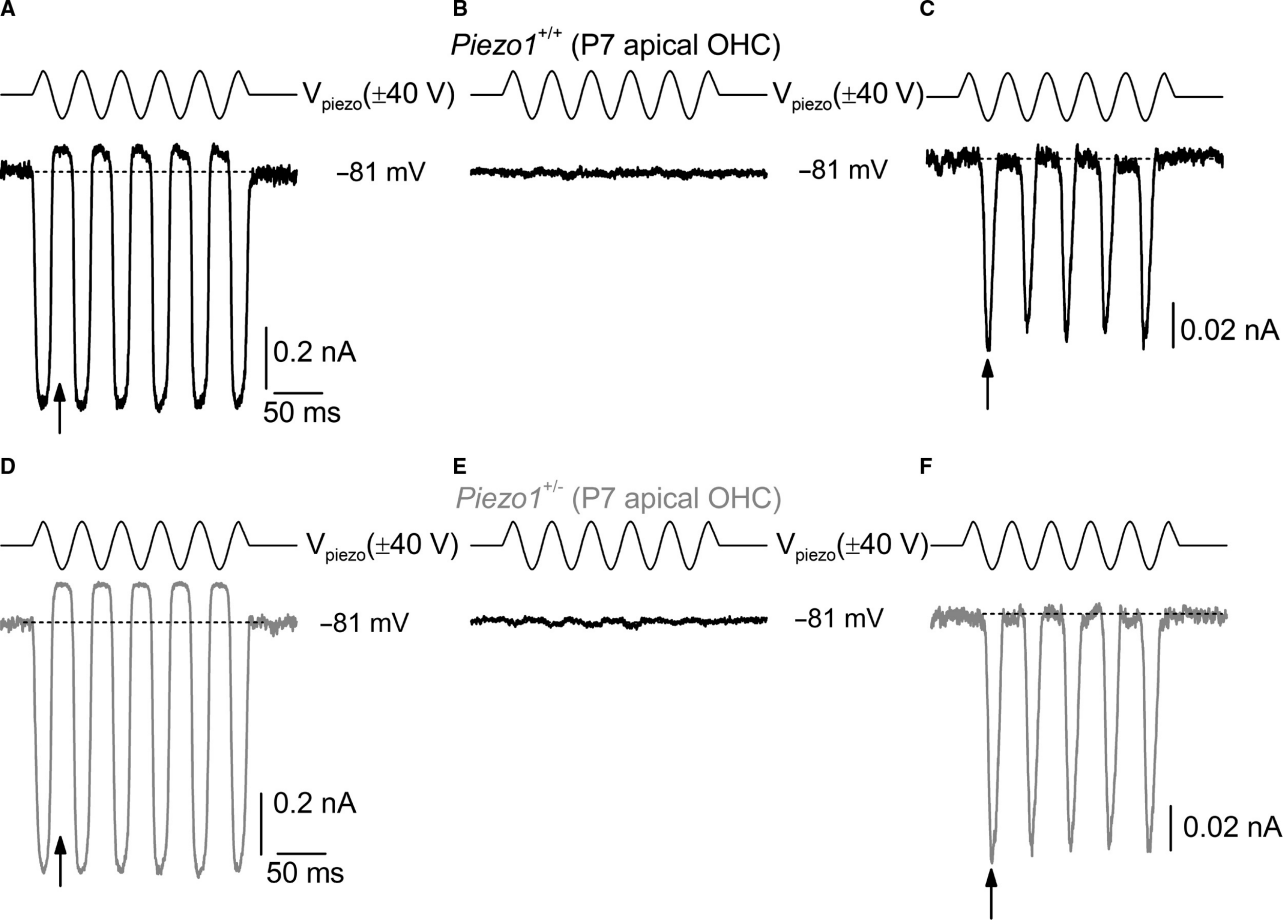
The anomalous mechanoelectrical transducer (MET) current is present in *Piezo1* ^+/−^ mice. A–F, MET currents recorded from apical P7 outer hair cells (OHCs) of *Piezo1* ^*+/+*^ (A–C) and *Piezo1* ^+/−^ (D–F) mice with an intact hair bundle (A and D), after being damaged by repeated strong stimulation (B and E) and obtained by applying strong mechanical stimulation to the cuticular plate and the damaged hair bundle (C and F), which has previously been used to elicit the anomalous MET current (Marcotti et al. 2014). Note that the anomalous MET current is obtained using negative stimuli (C and F: arrows), which normally closes the MET current available at rest (A and D: arrows).

The Piezo1 channel responds to sheer stress caused by frictional force (Bae et al. 2013; Li et al. 2014; Ranade et al. 2014a). Therefore, we tested whether the anomalous MET current present in wild‐type cochlear hair cells (Beurg et al. 2014; Marcotti et al. 2014) was affected in *Piezo1* ^+/−^ mice. This anomalous current is not tip link mediated and can be elicited under a variety of conditions (Marcotti et al. 2014), including mechanical overstimulation of the cuticular plate. We recorded MET currents from apical coil OHCs of both *Piezo1* ^+/+^ and *Piezo1* ^+/−^ animals before (Fig. 4A, D) and after (Fig. 4B, E) irreversible damage to the hair bundles by overstimulation. After this procedure, we were able to record the anomalous currents in both *Piezo1* ^+/+^ and *Piezo1* ^+/−^ OHCs by applying strong sinusoidal stimuli with a fluid jet positioned in close proximity to the cuticular plate and the damaged hair bundle (Fig. 4C, F). While the normal MET current was elicited during positive driver voltages (e.g., excitatory bundle deflections: arrow in Fig. 4A, D), the anomalous current was obtained during the negative phase of the sinewave stimulus, which is normally associated with inhibitory responses (arrow in Fig. 4C, F). We found that the amplitude of the anomalous current at 81 mV was similar between *Piezo1* ^+/+^ (−102 ± 44 pA, *n *=* *3) and *Piezo1* ^+/−^ (−87 ± 12 pA, *n *=* *5) OHCs.

## Discussion

In this work, we found no evidence for profound hearing loss in *Piezo1* ^+/−^ mice, and more specifically, we established that the size of the MET current and its biophysical properties such as Ca^2+^‐dependent adaptation and resting open probability were similar between *Piezo1* ^+/+^ and *Piezo1* ^+/−^ OHCs, which show haploinsufficiency for the protein (Li et al. 2014). Moreover, the recently described anomalous current activated by sheer force exerted onto the cuticular plate (Beurg et al. 2014; Marcotti et al. 2014) was unaffected by *Piezo1* haploinsufficiency.

The full molecular identity of the MET channel in mammalian cochlear hair cells is still unknown. TMC1 and TMC2 are candidate subunits based on several lines of evidence (Kawashima et al. 2011; Pan et al. 2013; Corns et al. 2016) including the fact that they are required for mechanotransduction in hair cells (Kawashima et al. 2011). TMC1 and TMC2 have also been shown to localize at the tip of the shorter stereocilia (Kurima et al. 2015) where the MET channels are located (Beurg et al. 2009), can restore sensory transduction when exogenously expressed in mice that are deficient of such proteins (Askew et al. 2015), and in humans mutations in *TMC1* cause dominant progressive hearing loss (DFNA36) and recessive profound congenital deafness (DFNB7/B11) (Kurima et al. 2002). However, recent studies have shown that an additional mechano‐activated current is present in TMC1/TMC2 double knockouts (Beurg et al. 2014). This anomalous current is elicited by applying unphysiologically strong mechanical stimuli eliciting a sheer force displacement onto the cuticular plate of hair cells (Beurg et al. 2014; Marcotti et al. 2014). The presence of this current has led to the suggestion that the channels underlying it could be the MET channel precursor in cochlear hair cells, although the biophysical and pharmacological properties of the anomalous channel are somewhat different from those of the normal MET current (Marcotti et al. 2014).

Piezo1 and Piezo2 are part of a conserved family of nonselective cation channels that are activated by mechanical stimuli (Coste et al. 2010, 2012) and have no sequence similarity with any other channel (Coste et al. 2010; Nilius and Honoré 2012). Expression profiles of mRNA for *Piezo1* and *Piezo2* are found in numerous mechanosensitive tissues (Coste et al. 2010). While Piezo2 is required for touch sensation in the mouse (Ranade et al. 2014b; Woo et al. 2014) and is associated with rapidly adapting mechanically stimulated currents in neurons of the dorsal root ganglion (Coste et al. 2010; Ranade et al. 2014b), Piezo1 is expressed in endothelial cells and red blood cells where it detects frictional force (sheer stress) (Bae et al. 2013; Li et al. 2014; Ranade et al. 2014a). Moreover, *Piezo1* (*Fam38A)* has been identified in the transcriptomes of cochlear hair cells (Liu et al. 2014). Piezo proteins form a homotetramer of about 1.2 MDa and reconstitution experiments of *Piezo1* in the lipid bilayer resulted in the recording of a spontaneous cation current, indicating that it is able to form a channel‐forming protein (Coste et al. 2012). The ability of an E2133K point mutation in Piezo1 to modify the channel unitary conductance, Ca^2+^ permeability, and the block potency of the polycationic blocker ruthenium red (Coste et al. 2015), suggests that Piezo1 constitutes a pore‐forming subunit. This was confirmed by the recently described structure of Piezo1, which has highlighted a central pore module (Ge et al. 2015). Our data demonstrated that despite Piezo1 being an excellent candidate for mediating mechanosensory transduction in cochlear hair cells, *Piezo1* haploinsufficiency did not alter any of the biophysical properties of the MET current recorded from apical or basal cochlear OHCs nor in the anomalous current activated by sheer stimuli.

## Conclusion

Our findings show that *Piezo1* haploinsufficiency (*Piezo1* ^+/−^) has no functional consequences on the MET current (Figs. 1, 2, 3) and the tip‐link independent anomalous current activated by sheer force (Fig. 4), indicating that Piezo1 is unlikely to be a crucial component of the MET apparatus in the mammalian auditory system.

## Conflict of Interest

The authors declare no conflict of interest.
